# Supplementary material for: The mediating role of internal motivation on the relationship between ethical leadership and employee performance in hospitals in Northern Jordan
Source: PLoS One. 2026 Jan 16;21(1):e0341065. doi: 10.1371/journal.pone.0341065 (PMC12810833; doi:10.1371/journal.pone.0341065)

**(Kolmogorov-Smirnov Test)/Q-Q Plots**

**Ethical Leadership**

**
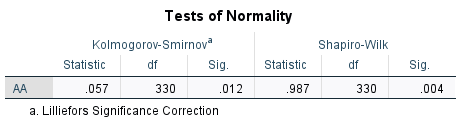
**

**
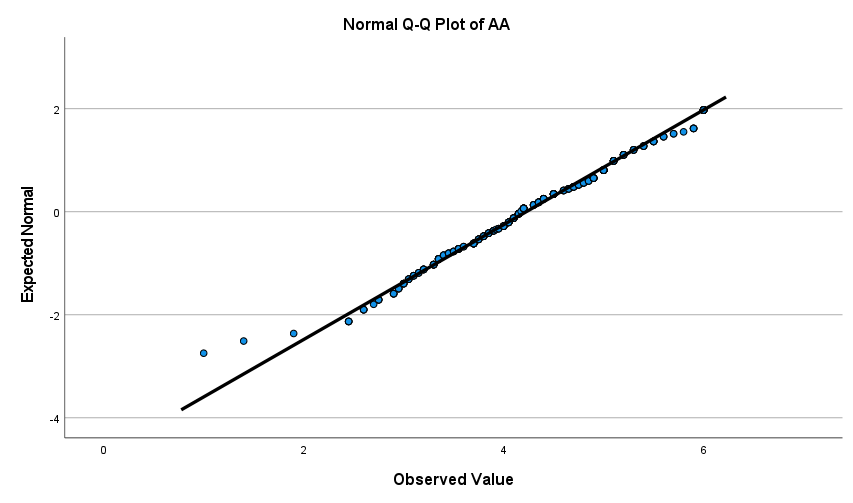
**

**Employee Performance**

**
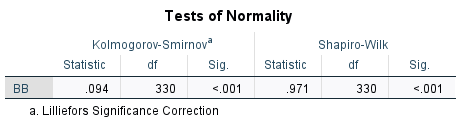
**

**
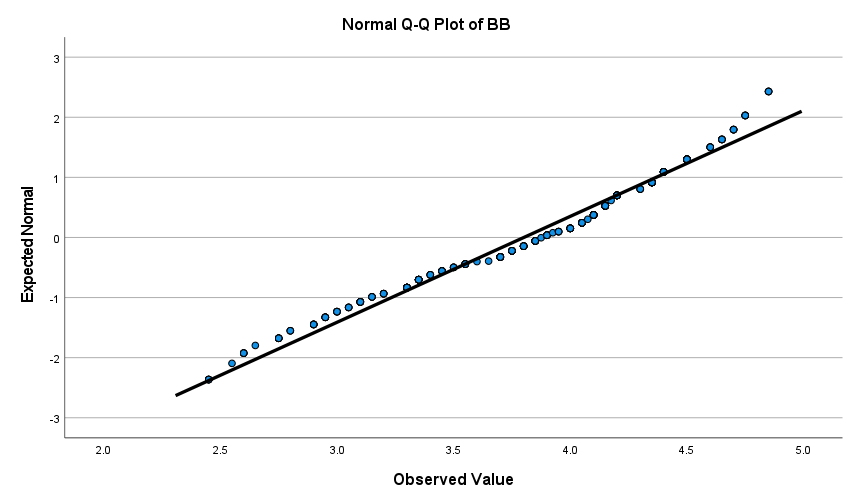
**

**Internal Motivation**

| **Tests of Normality** | | | | | | |
| --- | --- | --- | --- | --- | --- | --- |
|  | Kolmogorov-Smirnov^a^ | | | Shapiro-Wilk | | |
|  | Statistic | df | Sig. | Statistic | df | Sig. |
| CC | .112 | 330 | .000 | .949 | 330 | .000 |
| a. Lilliefors Significance Correction | | | | | | |


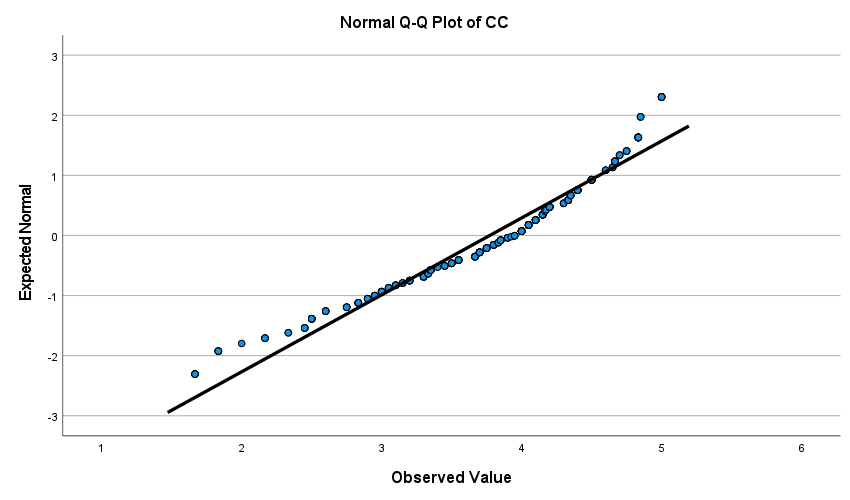

Supplement: S1 File — (DOCX) [file pone.0341065.s001.docx]
