# Supplementary material for: The mediating role of internal motivation on the relationship between ethical leadership and employee performance in hospitals in Northern Jordan
Source: PLoS One. 2026 Jan 16;21(1):e0341065. doi: 10.1371/journal.pone.0341065 (PMC12810833; doi:10.1371/journal.pone.0341065)
Supplement: S2 File — (DOCX) [file pone.0341065.s002.docx]

| **Model** | | **Collinearity Statistics** | |
| --- | --- | --- | --- |
|  |  | **Tolerance** | **VIF** |
| Ethical leadership | Employee performance | 1.00 | 1.00 |
| Internal motivation |  | 1.00 | 1.00 |
| Ethical leadership | Internal motivation | 1.00 | 1.00 |

**Table 2:** Results of The VIF test and The Tolerance test.
