# Supplementary material for: The mediating role of internal motivation on the relationship between ethical leadership and employee performance in hospitals in Northern Jordan
Source: PLoS One. 2026 Jan 16;21(1):e0341065. doi: 10.1371/journal.pone.0341065 (PMC12810833; doi:10.1371/journal.pone.0341065)
Supplement: S3 File — (DOCX) [file pone.0341065.s003.docx]

| **Variable** | **Category** | **Frequency** | **Percentage** |
| --- | --- | --- | --- |
| **Age** | Less than 25 years | 48 | 14.5 |
|  | 25-=35 years | 124 | 37.6 |
|  | 36-45 years | 97 | 29.4 |
|  | Over 45 years | 61 | 18.5 |
| **Gender** | Male | 105 | 31.8 |
|  | Female | 225 | 68.2 |
| **Marital status** | Single | 95 | 28.8 |
|  | Married | 227 | 68.8 |
|  | Divorcee | 6 | 1.8 |
|  | Widower | 2 | 0.6 |
| **Education level** | Diploma | 95 | 28.8 |
|  | Bachelor's | 190 | 57.6 |
|  | Masters | 43 | 13.0 |
|  | PhD | 2 | 0.6 |
| **Organization ownership** | University | 90 | 27.3 |
|  | Public | 130 | 39.4 |
|  | Private | 110 | 33.3 |
| **Work experience** | Less than 2 years | 67 | 20.3 |
|  | 2-5 years | 60 | 18.2 |
|  | 6-10 years | 50 | 15.2 |
|  | 11-20 years | 87 | 26.4 |
|  | 20-Over 21 years | 66 | 20.0 |
| **Job category** | Clinical employee | 175 | 53.0 |
|  | Administrative employee | 155 | 47.0 |
| **Total** |  | **330** | **100.0** |

**Table 3:** Employee Characteristics for Clinical and Administrative Employees in The Selected Hospitals
