# Supplementary material for: The mediating role of internal motivation on the relationship between ethical leadership and employee performance in hospitals in Northern Jordan
Source: PLoS One. 2026 Jan 16;21(1):e0341065. doi: 10.1371/journal.pone.0341065 (PMC12810833; doi:10.1371/journal.pone.0341065)
Supplement: S4 File — (DOCX) [file pone.0341065.s004.docx]

**Table 4:** Descriptive Analysis (Mean and Standard Deviation) of Ethical Leadership Items

| **Number** | **Item Name** | **Mean scores** | **Standard Deviation** | **Importance** | **Level** |
| --- | --- | --- | --- | --- | --- |
| **1** | **My supervisor shows a strong concern for ethical and moral values** | 4.85 | 1.04 | 1 | High |
| **2** | **My supervisor communicates clear ethical standards for members** | 4.67 | 1.08 | 2 | High |
| **8** | **My supervisor regards honesty and integrity as important personal values** | 4.63 | 1.15 | 3 | High |
| **10** | **My supervisor holds members accountable for using ethical practices in their work** | 4.56 | 1.18 | 4 | High |
| **3** | **My supervisor sets an example of ethical behavior in his or her decisions/ actions** | 4.54 | 1.22 | 5 | High |
| **4** | **My supervisor is honest and can be trusted to tell the truth** | 4.53 | 1.26 | 6 | High |
| **9** | **My supervisor opposes the use of unethical practices to increase performance** | 4.51 | 1.32 | 7 | High |
| **5** | **My supervisor keeps his or her actions consistent with his or her stated values (walks the talk)** | 4.48 | 1.25 | 8 | High |
| **7** | **My supervisor insists on doing what is ethical even when it is not easy** | 4.37 | 1.26 | 9 | High |
| **6** | **My supervisor can be trusted to carry out promises and commitments** | 4.31 | 1.28 | 10 | Medium |
| **Total** | **Ethical Leadership** | **4.55** | **0.90** | --- | **High** |

**Table 5:** Descriptive Analysis (Mean and Standard Deviation) of Internal Motivation Items (N=6)

| **Number** | **Item Name** | **Mean scores** | **Standard deviation** | **Importance** | **Level** |
| --- | --- | --- | --- | --- | --- |
| **6** | **If choosing between two jobs, the most important criterion is ‘which is more fun** | 4.05 | 1.19 | 1 | High |
| **2** | **If I did not enjoy doing my job at work, I would leave** | 3.97 | 1.19 | 2 | High |
| **1** | **I only like to do things that are fun** | 3.90 | 1.16 | 3 | High |
| **3** | **I often put off work so that I can do something else that is more fun** | 3.83 | 1.33 | 4 | High |
| **4** | **When choosing jobs, I usually choose the one that sounds like the most fun** | 3.79 | 1.40 | 5 | High |
| **5** | **The people I choose to spend my time with are the most fun to be with** | 3.78 | 1.33 | 6 | High |
| **Total** | **Internal motivation** | **3.89** | **0.78** | **---** | High |

**Table 6:** Descriptive Analysis (Mean and Standard Deviation) of Employee Performance Items (N=20)

| **Number** | **Item Name** | **Mean scores** | **Standard deviation** | **Importance** | **Level** |
| --- | --- | --- | --- | --- | --- |
| **18** | **Great deal of time spent with personal phone conversations** | 4.36 | 0.92 | 1 | High |
| **16** | **Gives advance notice when unable to come to work** | 4.27 | 1.03 | 2 | High |
| **17** | **Takes undeserved work break** | 4.25 | 1.02 | 3 | High |
| **1** | **Adequately completes assigned duties** | 4.15 | 0.92 | 4 | High |
| **20** | **Adheres to informal rules devised to maintain order** | 3.85 | 1.19 | 5 | High |
| **14** | **Passes along information to co-workers** | 3.85 | 0.96 | 6 | High |
| **8** | **Helps others who have been absent** | 3.76 | 0.94 | 7 | High |
| **7** | **Fails to perform essential duties** | 3.75 | 1.03 | 8 | High |
| **19** | **Complains about insignificant things at work** | 3.75 | 1.17 | 9 | High |
| **6** | **Neglects aspects of the job he/she is obligated to perform** | 3.72 | 1.05 | 10 | High |
| **4** | **Meets formal performance requirements of the job** | 3.72 | 1.04 | 11 | High |
| **9** | **Helps others who have heavy workloads** | 3.72 | 0.91 | 12 | High |
| **15** | **Attendance at work is above the norm** | 3.71 | 0.90 | 13 | High |
| **11** | **Takes time to listen to co-workers' problems and worries** | 3.69 | 0.88 | 14 | High |
| **10** | **Assists supervisor with his/her work (when not asked)** | 3.66 | 0.85 | 15 | Medium |
| **13** | **Takes a personal interest in other employees** | 3.66 | 0.89 | 16 | Medium |
| **2** | **Fulfills responsibilities specified in job description** | 3.60 | 1.24 | 17 | Medium |
| **5** | **Engages in activities that will directly affect his/her performance evaluation** | 3.59 | 1.18 | 18 | Medium |
| **3** | **Performs tasks that are expected of him/her** | 3.58 | 1.20 | 19 | Medium |
| **12** | **Goes out of way to help new employees** | 3.34 | 1.09 | 20 | Medium |
| **Total** | **Employee performance** | **3.80** | **0.57** | --- | High |
