# Supplementary material for: The mediating role of internal motivation on the relationship between ethical leadership and employee performance in hospitals in Northern Jordan
Source: PLoS One. 2026 Jan 16;21(1):e0341065. doi: 10.1371/journal.pone.0341065 (PMC12810833; doi:10.1371/journal.pone.0341065)
Supplement: Attachment — Submitted filename: Point by Point Response to Reviewers.docx [file pone.0341065.s007.docx]

| **The MEDIATING ROLE OF INTERNAL MOTIVATION ON THE RELATIONSHIP BETWEEN ETHICAL LEADERSHIP AND EMPLOYEE PERFORMANCE IN HOSPITALS IN NORTHERN JORDAN** | | |  |
| --- | --- | --- | --- |
| First of all, we would like to thank the editor and reviewers for their highly efforts and time in reviewing our manuscript. We are highly appreciated your valuable comments and feedback. We just worked hard to address all your comments and we wish that we addressed them effectively. | | |  |
| **Reviewers’ Comments** | | |  |
| Reviewer #1 | **Reviewer’s comments** | **Responses** |  |
|  | “Sampling Bias (Page 16): The use of convenience sampling may limit generalizability. Consider acknowledging this limitation and discussing strategies to mitigate bias (e.g., stratified sampling in future studies). | Addressed in the “study limitation” section  Page 27 (Line 613-615) |  |
|  | “The design precludes causal inferences. Clarify this limitation and recommend longitudinal studies to establish causality.” | Addressed in the “study limitation” section  Page 27 (Line 615-618) |  |
|  | “While scales like the Ethical Leadership Scale are cited, no evidence is provided for their validity in Jordanian healthcare contexts. Include pilot testing results or cultural adaptation details” | Done. We have added details about the pilot study  Page 11 (Line 302-303) |  |
|  | “Report effect sizes (e.g., Cohen’s *d*, R²) alongside *p*-values to assess practical significance (e.g., Table 4–7).” | Done. We have also added this information to the Results section alongside the tables. Tables 4–7 have been updated to include effect sizes in addition to p-values. Specifically, R² and Adjusted R² are reported for all models, and Cohen’s f² is provided along with the statistical significance (p-values. Page 19-23 |  |
|  | “The manuscript briefly mentions Jordan’s healthcare challenges but does not deeply contextualize how ethical leadership operates in high-stress, resource-constrained hospital environments. Expand using literature on nursing/healthcare leadership.” | Done. we have expanded the discussion and incorporated relevant literature on **nursing and healthcare leadership**  Page 4-5 (Line 39-55) |  |
|  | “The discussion of how internal motivation mediates the relationship is superficial. Link findings to psychological constructs like psychological safety or organizational identification (see recommended studies below).” | Done. We have expanded the discussion of internal motivation as a mediator by explicitly linking our findings to relevant psychological constructs  Page 5 (Line 56-62) |  |
|  | “VIF values of 1.00 (Page 19) are unusually low. Verify calculations and ensure multicollinearity diagnostics are accurately reported.” | We have double-checked the calculations, and the VIF values of 1.00 are correct |  |
|  | “The Kolmogorov-Smirnov test is sensitive to large samples. Supplement with visual checks (e.g., Q-Q plots) in supplementary files.” | Done. Q–Q plots for all main study variables have been added to the supplementary file (S.F.1) |  |
|  | “The statement “All relevant data are within the manuscript and its Supporting Information files” conflicts with the sample size of 330. Clarify if raw data (e.g., anonymized responses) are included in supplementary files” | Done. The statement regarding data accessibility was revised  Page 29 (Line 717) |  |
|  | “Use consistent notation (e.g., H??, H??).” | Done. We have revised the hypotheses’ notations  Page 8 (Line 224) |  |
|  | “Label predictors and outcomes clearly (e.g., “Ethical Leadership → Internal Motivation.” | Done. We have added notes to clearly indicate the predictors and outcomes for all hierarchical regression analyses |  |
|  | “Revise awkward phrasing (e.g., Page 10: “Data was gathered from 330 clinical and administrative employees from February to March 2024 using convenience sampling. from five hospitals...” | Done. Page 2 (Line 5) |  |
| Reviewer #2 | **Reviewer’s comments** | **Responses** |  |
|  | “Update research articles for updated years.” | Done. We have updated the manuscript to include the most recent and relevant research articles  Page 4-7 (Line 38-223) |  |
|  | Formatting issues | Done. We have carefully revised the manuscript to correct all formatting issues |  |
|  | Hypothesis table required | Done. A table summarizing all study hypotheses has been added to the manuscript  Page 8 (Line 224) |  |
|  | Give more clarity to results discussion | Done. In the revised manuscript, we have clarified the discussion of the results by more explicitly linking key findings to the hypotheses and existing literature.  Page 25-26 (Line 491-611) |  |
|  | Literature review is missing and support of articles given in discussion and conceptual model draw after results | Done. We have revised the literature review and discussion sections, ensuring that all key findings are supported by relevant studies. |  |
| Reviewer #3 | **Reviewer’s comments** | **Responses** |  |
|  | “Incorrect Hypothesis Formulation” | Done. The hypotheses have been revised to ensure correct formulation  Page 8 (Line 224) |  |
|  | “Weak Theoretical Foundation Each hypothesis lacks grounding in prior empirical evidence. Action Required: Cite 2–3 key studies per hypothesis to justify expected relationships (e.g., "Consistent with Brown et al. (2005) and Walumbwa et al. (2011), we posit that..."). | Done. We have strengthened the theoretical foundation in the literature review by explicitly grounding each hypothesis in prior empirical evidence  Page 4-7 (Lines 39-244) |  |
|  | Incomplete Scale Documentation Scoring methods are unclear (e.g., interpretation of Likert-scale means). | Done. The scoring methods and interpretation of Likert-scale means for all study variables were added.  Page 10 (Lines 284-287)  Page 11 (Lines 291-293)  Page 11 (Lines 298-300) |  |
| Editor | **Editor’s comments** |  |  |
|  | Clarify directionality: "Higher item scores indicate stronger employee performance (EP)." Provide example items (e.g., "Sample EP item: 'I consistently exceed performance targets.'").  Report Composite Reliability (CR > 0.7) and Average Variance Extracted (AVE > 0.5) for validity. | Done. We have clarified the instruments’ directionality and reported their reliability and validity.  Page 10 (Lines 284-287)  Page 11 (Lines 291-293)  Page 11 (Lines 298-300)  Page 11 (Lines 304-306) |  |
|  | Descriptive statistics (means, SDs, correlations) must precede regression/SEM. | Done. The descriptive statistics, including means, standard deviations, and correlations, are presented prior to the regression analyses. |  |
|  | Critical Issue: The null hypotheses cannot support the final model. Use: Bootstrap mediation analysis (PROCESS/AMOS) with 95% CIs for indirect effects. SEM fit indices (CFI/RMSEA) if structural paths are tested. | Done. We conducted a bootstrapping mediation analysis in AMOS with 5,000 bias-corrected samples. All hypothesized paths were significant, and the 95% confidence interval for the indirect effect excluded zero, supporting partial mediation.  Page 22-23 (Lines 441-451) |  |
|  | Discussion Lacks Hypothesis-Level Analysis Contrast results with prior work for each hypothesis (e.g., "Contrary to Javed et al. (2017), our findings show..."). | Done. We have revised the Discussion section to provide hypothesis-level analysis.  Page 25-26 (Lines 491-611) |  |
|  | Missing "Limitations" Section | We have added “study limitation” section to the revised manuscript.  Page 27 (Lines 612-623) |  |
|  | Sampling bias (convenience sampling). | Addressed in the limitation section  Page 27 (Lines 613-615) |  |
|  | Cross-sectional design (causality cannot be inferred). | Addressed in the limitation section  Page 27 (Lines 615-618) |  |
|  | Context specificity (Jordanian healthcare only). | We have added a statement in the limitations section to clarify this context specificity and suggest that future research replicate the study in different countries or healthcare systems to enhance external validity  Page 27 (Lines 618-623) |  |
|  | Overall Quality Concerns The paper suffers from unclear logic (e.g., mismatched hypotheses/model) and requires extensive restructuring. Recommendation: Study high-quality exemplars (e.g., Academy of Management Journal articles on ethical leadership) | We reviewed key Academy of Management Journal articles on ethical leadership (e.g., Yen, 2023; Wang, 2024) and restructured the manuscript to better align hypotheses with analyses and improve overall coherence |  |
