# Supplementary material for: The mediating role of internal motivation on the relationship between ethical leadership and employee performance in hospitals in Northern Jordan
Source: PLoS One. 2026 Jan 16;21(1):e0341065. doi: 10.1371/journal.pone.0341065 (PMC12810833; doi:10.1371/journal.pone.0341065)
Supplement: Attachment — Submitted filename: Point_by_Point_Response_to_Reviewers_auresp_2.docx [file pone.0341065.s008.docx]

| **The MEDIATING ROLE OF INTERNAL MOTIVATION ON THE RELATIONSHIP BETWEEN ETHICAL LEADERSHIP AND EMPLOYEE PERFORMANCE IN HOSPITALS IN NORTHERN JORDAN** | | | |
| --- | --- | --- | --- |
| First of all, we would like to thank the editor and reviewers for their highly efforts and time in reviewing our manuscript. We are highly appreciated your valuable comments and feedback. We just worked hard to address all your comments and we wish that we addressed them effectively. | | | |
| **Reviewers’ Comments** | | | |
| Reviewer #2 | **Reviewer’s comments** | | **Responses** |
|  | “Revise Data Analysis chapters: Having errors like regression analysis for demographics and most of variable are insignificant.no discussion regarding this.clarity of results is missing.” | | Done. The Results and discussion sections have been revised. Additional explanatory text has been added to improve clarity and interpretation of the regression results.  Page 13 (Line 390-394)  Page 17 (Line 446)  Page 18 (Line 461-462)  Page 23 (Line 517-520) |
|  | “Adopt professional reading format for readers interests e.g in sum,authors highlights,it would be passive voice and n Researcher tone like we,you,i words..” | | Done. We have carefully revised the manuscript to adopt a more professional and academic reading format |
| Reviewer #4 | **Reviewer’s comments** | | **Responses** |
|  | “The manuscript is comprehensive but lengthy especially the Literature Review. Consider reducing repetition and focusing on the most relevant studies to improve readability.” | | Done. We have revised the manuscript to reduce repetition and streamline the Literature Review  Page 4-5 (Line 34-140) |
|  | The conclusion would benefit from more specific and actionable recommendations for hospital managers and policymakers based on the study findings | | Done. We have revised the conclusion to include more specific and actionable recommendations for hospital managers and policymakers, directly derived from the study findings  Page 27 (Line 577-581) |
|  | Ensure consistency in terminology (e.g., “internal motivation” vs. “intrinsic motivation”) and headings. | We have carefully reviewed the manuscript to ensure consistency in terminology, using “internal motivation” uniformly throughout | |
